# Low-cost programmable sine wave driver for inductive loads with built-in current sensing

**DOI:** 10.1016/j.ohx.2026.e00815

**Published:** 2026-07-24

**Authors:** Michal Labuda, Veronika Wohlmuthova, Ladislav Janousek

**Affiliations:** University of Zilina, Faculty of Electrical Engineering and Information Technology, Department of Electromagnetic and Biomedical Engineering, Univerzitna 8215/1, Zilina 010 26, Slovakia

**Keywords:** Sinusoidal waveform generation, Direct Digital Synthesis, Inductive load excitation, High-current operational amplifier

## Abstract

This paper presents the design and implementation of a compact and versatile device for generating and amplifying sinusoidal waveforms to drive inductive loads. The system is built around the AD9833 programmable waveform generator and the OPA548 high-current operational amplifier, forming a flexible platform capable of producing high-power sine wave excitation signals. The device can operate in two modes: internal signal generation using the AD9833 direct digital synthesis (DDS) module, or external signal input from a laboratory function generator, providing increased adaptability for various applications. To enable real-time monitoring and protection, an ACS724LLCTR-2P5AB-S Hall-effect current sensor is integrated into the output stage for accurate measurement of load current. The proposed design offers a cost-effective solution for applications requiring controlled excitation of inductive elements such as coils, transformers, or electromagnetic actuators. Experimental testing confirms the ability of the system to deliver stable amplified sine waves with reliable current sensing, making it suitable for laboratory experimentation, educational purposes, and small-scale industrial testing environments.

## Graphical abstract

**Figure:**
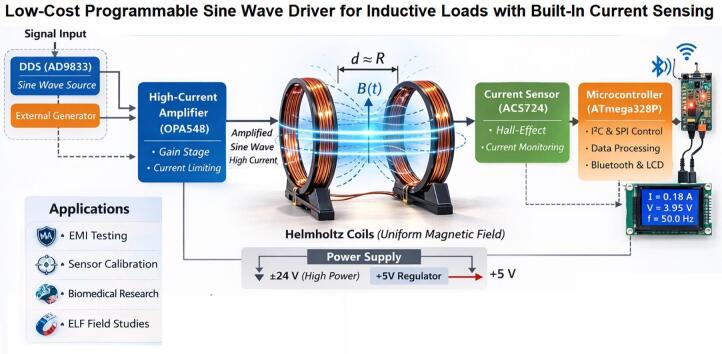

## Specifications table

**Table:** 

Hardware name	*Programmable Sine Wave Driver for Inductive Loads with Built-In Current Sensing*
Subject area	Educational tools and open source alternatives to existing infrastructure
Hardware type	Electrical engineering and computer science
Closest commercial analog	*AFG-3031GW INSTEK-Laboratory generator arbitrary+* *WA301AIM-TTI – power amplifier*
Open source license	CC BY 4.0
Cost of hardware	117.05 €
Source file repository	*https://data.mendeley.com/datasets/7wmwr9sch9/1*

## Hardware in context

**Fig. 1:**
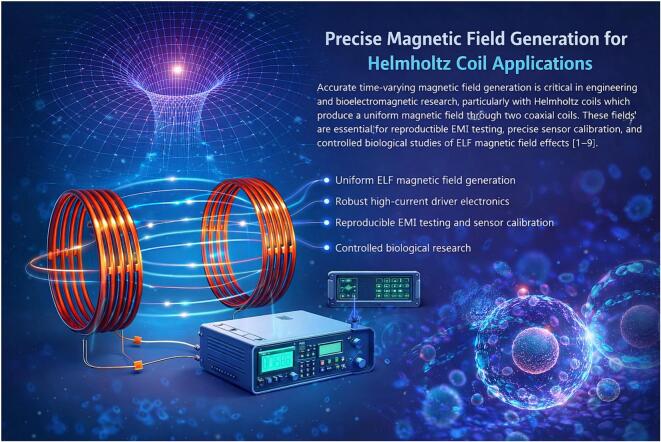
Integrated system for sinusoidal signal generation, power amplification, and uniform magnetic field production.

Precise generation of time-varying electromagnetic fields is essential in applications such as electromagnetic interference (EMI) testing, sensor calibration, and bioelectromagnetic research. Helmholtz coils, consisting of two coaxial coils separated by a distance equal to their radius, are widely used due to their ability to produce a uniform magnetic field in the central region [1]. Achieving stable and reproducible field conditions requires accurate control of the excitation current supplied to inductive loads, Fig. 1.

In biological research, extremely low frequency (ELF) magnetic fields are used to investigate cellular and tissue responses [2], [3], [4], [5]. These studies demonstrate that ELF exposure can influence cell proliferation, cytoskeletal dynamics, and tissue regeneration, and that the response can be frequency-dependent [2], [3], [4], [5], [6], [7], [8], [9]. Since the generated magnetic field is directly proportional to the current flowing through the excitation coil, accurate control and monitoring of the load current are essential for reliable experimental conditions [3].

Conventional laboratory setups for generating such fields typically consist of a waveform generator, a power amplifier, and separate measurement instrumentation. While these solutions provide high performance, they are often costly, require complex configuration, and lack integration.

The hardware presented in this work addresses these limitations by integrating programmable sinusoidal signal generation, high-current amplification, and real-time current sensing into a single low-cost platform. The system supports both internally generated signals using a direct digital synthesis (DDS) circuit and externally supplied signals from a laboratory generator, providing flexibility for different experimental scenarios.

The main contribution of this work is the open-hardware integration of signal generation, power amplification, and current sensing into a single device optimized for ELF applications. This approach reduces system complexity and cost while maintaining adequate performance for laboratory experimentation, educational use, and small-scale testing of inductive loads.

## 2 Hardware description

**Fig. 2:**
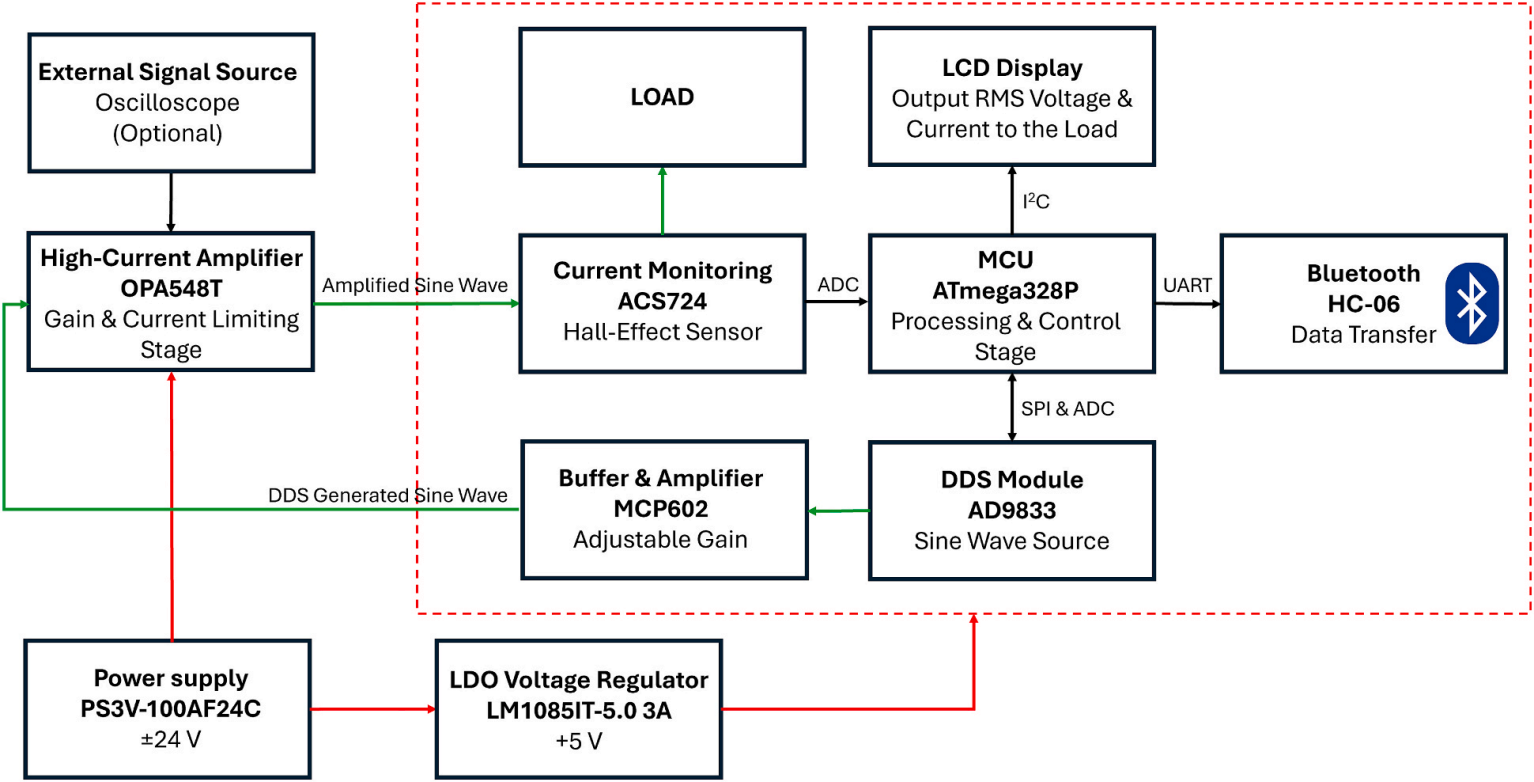
Block diagram of the proposed device.

The designed device (Fig. 2) integrates signal generation, amplification, and current measurement for driving inductive loads in the ELF range (up to 300 Hz). The system consists of five main hardware stages: signal generation, power amplification, current sensing, control and communication, and power supply.

The signal generation stage provides two selectable input sources. The first is an internal sinusoidal generator based on the AD9833 DDS integrated circuit (Analog Devices, Massachusetts, USA), which enables precise frequency control with high resolution and stable waveform generation. The DDS output is buffered and conditioned using low-noise operational amplifiers to remove DC offset and adjust amplitude before further processing. The second option allows connection of an external laboratory signal generator. A jumper selector is used to switch between internal and external sources, providing flexibility for different laboratory and testing applications.

The core of the system is the high-current amplification stage built around the OPA548 operational amplifier (Texas Instruments, Texas, USA). The amplifier is configured in a non-inverting topology and powered from a symmetrical ± 24 V supply. It provides sufficient voltage and current capability to drive inductive loads such as coils, transformers, or electromagnetic actuators. The gain is preset but can be adjusted by modifying selected passive components. The amplifier also includes adjustable current limiting, allowing safe operation under different load conditions. Fast-recovery protection diodes are placed at the output to protect against voltage spikes caused by inductive switching.

Load current monitoring is implemented using the ACS724 Hall-effect current sensor (Allegro MicroSystems, New Hampshire, USA). The sensor provides galvanic isolation and outputs a voltage proportional to the instantaneous current flowing through the load. Signal conditioning circuitry, including buffering and band-pass filtering, limits the measurement bandwidth to the ELF range while suppressing high-frequency interference. This stage enables current measurement up to ± 2.5 A without significantly affecting the load circuit.

System control is implemented using an ATmega328P microcontroller (Microchip Technology, Arizona, USA). To maintain low system cost and simplified hardware integration, the current sensing stage utilizes the internal 10-bit ADC of the microcontroller together with the ACS724 Hall-effect sensor, resulting in moderate measurement accuracy that remains sufficient for the intended ELF laboratory applications. The microcontroller communicates with the DDS generator via the SPI interface to adjust the output frequency and processes the measured current signal to compute RMS values. Measurement results are displayed on an LCD module using the I^2^C interface. A Bluetooth module enables wireless transmission of data to a computer, extending the usability of the device in laboratory environments.

The power supply stage consists of two switched-mode power supplies configured to provide a symmetrical ± 24 V rail for the power amplifier. A linear voltage regulator generates a stable + 5 V supply for digital and low-voltage analog circuitry. This separation improves stability and reduces interference between power and signal-processing stages.

Overall, the proposed hardware integrates signal generation, high-current amplification, measurement, and digital control into a single device suitable for laboratory experimentation and characterization of inductive loads.

Conventional laboratory setups for driving inductive loads typically require a combination of a waveform generator, a power amplifier, and a power supply. For example, a representative setup consisting of a Rigol DG4162 arbitrary waveform generator, a linear power amplifier (e.g., Hubert A1110-16), and a laboratory power supply results in a total system cost on the order of 1700–2500 €. In contrast, the proposed device integrates signal generation, amplification, and current sensing into a single platform with a total cost of 117.05 €, representing a significant reduction in system cost while maintaining sufficient performance for ELF applications, Table 1.

• The designed device is a low-cost alternative to existing power amplifiers and signal generators.
• Precise DDS-based sinusoidal generation ensures accurate and frequency-stable ELF magnetic field excitation.
• High-current OPA548 amplifier drives Helmholtz coils without significant waveform distortion.
• Real-time current monitoring guarantees accurate and reproducible magnetic field strength.
• Adjustable current limiting and protection circuitry enhance operational safety and reliability.
• Digital control and data logging support repeatable and well-documented biological and EMI experiments.

**Table 1:** Comparison between the proposed device and typical commercial setup.

**System**	**Signal Generation**	**Amplification**	**Current Sensing**	**Integration**	**Approx. Cost**
Proposed device	Integrated DDS	Integrated (OPA548)	Integrated (ACS724)	Single device	**117.05 €**
Commercial setup (DG4162 + A1110-16 + PSU)	External generator	External amplifier	External instruments	Multi-device	**1700–2500 €**

## 3 Design files summary

**Table:** 

**Design file name**	**File type**	**Open-source license**	**Location of the file**	**Version**
PCB_SWD	CAD Files	CC BY 4.0	https://data.mendeley.com/datasets/7wmwr9sch9/1	1.0
SWD.mlapp	Matlab Files	CC BY 4.0	https://data.mendeley.com/datasets/7wmwr9sch9/1	1.0
SWD_firmware	Microchip studio Files	CC BY 4.0	https://data.mendeley.com/datasets/7wmwr9sch9/1	1.0

The repository contains PCB design files in EAGLE format, including schematic and layout files. The firmware for the ATmega328P is provided as a Microchip Studio project, including all source code required for compilation and programming. MATLAB scripts and the graphical user interface application are included for data visualization and analysis. The repository is organized into separate directories for hardware, firmware, and software components to facilitate reproducibility and reuse.

## 4 Bill of materials summary

**Table:** 

**Designator**	**Component**	**Pieces**	**Cost per unit −currency**	**Total cost −** **currency**	**Source of materials**
PCB_device	PCB	5	0.4 €	2 €	https://jlcpcb.com/
PCB_illumination device	PCB	5	0.4 €	2 €	https://jlcpcb.com/
R1, R3, R4, R9	Resistor 10 kΩ	4	1.08 €	4.32 €	https://sinewavedriver.short.gy/naQDci
R10, R11	Resistor 100 kΩ	2	0.086 €	0.17 €	https://sinewavedriver.short.gy/MGFvTk
R5	Resistor 14.7 kΩ	1	0.086 €	0.086 €	https://sinewavedriver.short.gy/Mcxuve
R6	Resistor 33 kΩ	1	0.006 €	0.006 €	https://sinewavedriver.short.gy/f1qsez
R7	Resistor 57.6 kΩ	1	0.115 €	0.115 €	https://sinewavedriver.short.gy/JuUq21
C1 − C5, C7, C9, C15	Capacitor 10 µF	9	0.757 €	6.81 €	https://sinewavedriver.short.gy/PtKlKA
C10, C12	Capacitor 47 nF	2	0.568 €	1.14 €	https://sinewavedriver.short.gy/e9mz6x
C11, C17	Capacitor 4.7 nF	2	1.12 €	2.24 €	https://sinewavedriver.short.gy/iNx1BO
C13	Capacitor 100 nF	1	0.628 €	0.628 €	https://sinewavedriver.short.gy/oHr1ET
D1, D2	Diode MUR410	2	0.31 €	0.62 €	https://sinewavedriver.short.gy/Yl7pCF
Display	Display 20x4	1	20.25 €	20.25 €	https://sinewavedriver.short.gy/LIPE27
HC-06	Bluetooth module HC-06	1	21.80 €	21.80 €	https://sinewavedriver.short.gy/b4EXZ1
IC1	Power amplifier OPA548	1	16.42 €	16.42 €	https://sinewavedriver.short.gy/Mkyxij
IC2, IC3	Operational amplifier MCP602P	2	0.765 €	1.53 €	https://sinewavedriver.short.gy/6t5VfU
J1, J2	Screw pad TBC05-03–1-G-G_REVA	2	0.671 €	1.34 €	https://sinewavedriver.short.gy/AFshCV
JP1-JP9	Male pin header	1	0.5 €	0.5 €	https://sinewavedriver.short.gy/A1Z8j9
SW1	Switch SDA03H1BD	1	1.27 €	1.27 €	https://sinewavedriver.short.gy/Ew5p4e
SW2	Push button 1825910–6	1	0.112 €	0.112 €	https://sinewavedriver.short.gy/kaaRXV
U1	Voltage regulator LM1085IT-5.0	1	1.86 €	1.86 €	https://sinewavedriver.short.gy/5lKZBD
U3	Hall current sensor ACS724LLCTR-2P5AB-S	1	2.82 €	2.82 €	https://sinewavedriver.short.gy/v1b8uv
U4	Microcontroller ATmega328P	1	2.41 €	2.41 €	https://sinewavedriver.short.gy/Jraigj
VR1	Potentiometer 100kΩ 3266 W-1-103LF	1	2.65 €	2.65 €	https://sinewavedriver.short.gy/CJU6hb
X1, X2	Female BNC PCB connector R141426	2	8.01 €	16.02 €	https://sinewavedriver.short.gy/zn4QMq
AD9833	GY-AD9833 DDS Signal Generator Module	1	7.85 €	7.85 €	https://sinewavedriver.short.gy/8dov6t
**Total cost:**	117.05 €				

*All prices are exclusive of VAT and applicable duty charges.

## 5 Build instructions

The device is first built by ordering printed circuit boards (PCB). Once the boards are delivered, electronic components are soldered onto each PCB according to the corresponding circuit diagrams. After completing the assembly of both boards, the firmware is programmed into the ATmega328P microcontroller to ensure proper operation of the system.

Other necessary tools for assembly:

• Soldering iron
• Solder wire
• Multimeter
• Side cutters
• Atmel ICE Programmer (for ATmega328P programming) [10].

### 5.1 PCB assembly

**Fig. 3:**
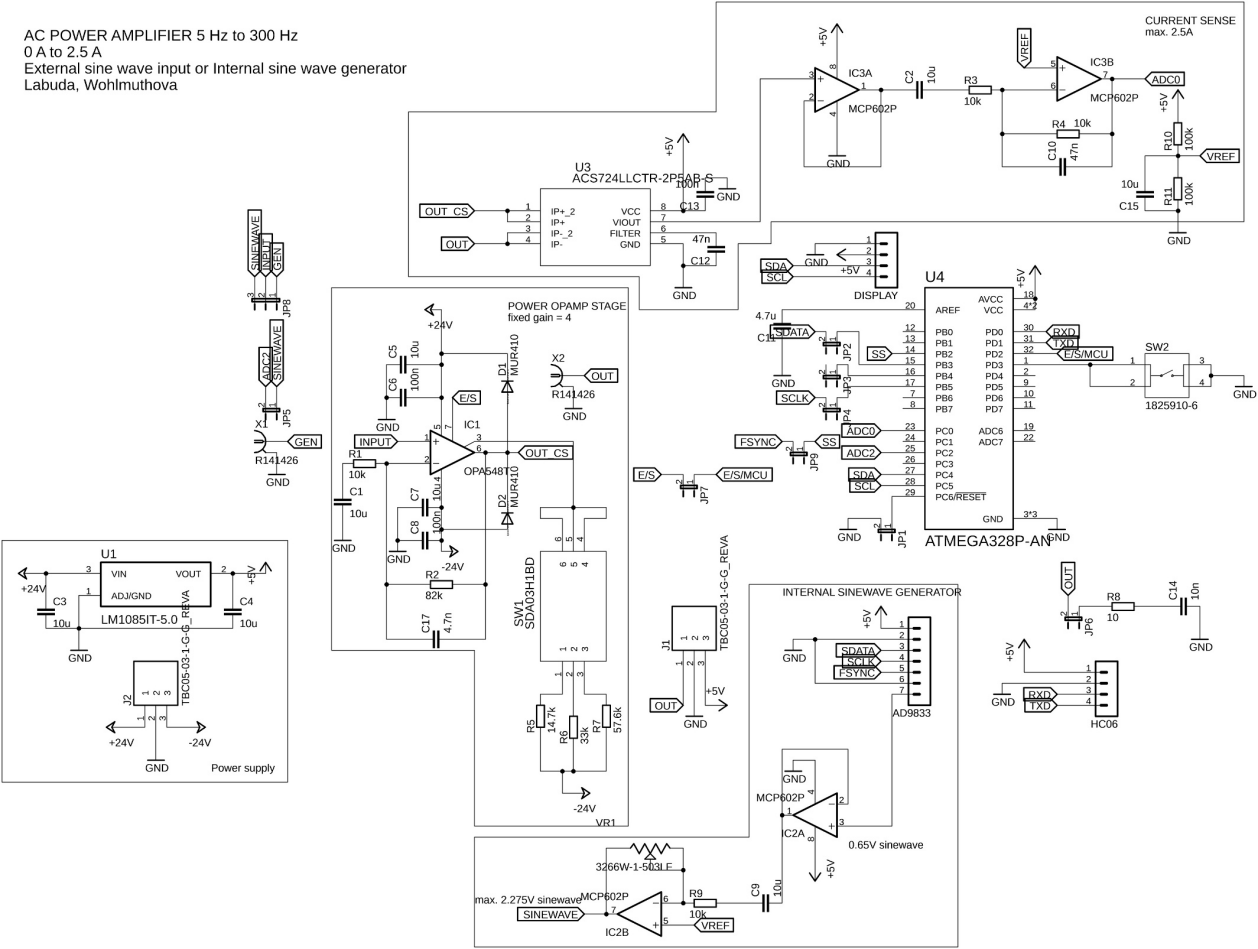
Device schematic created in EAGLE CAD Software.

The printed circuit boards were manufactured using the provided Gerber design files. Components were assembled according to the schematic shown in Fig. 3, following standard PCB assembly procedures. Assembly was performed progressively from low-profile passive components to integrated circuits, connectors, and larger mechanical parts. Particular attention should be paid to the orientation of polarized components and integrated circuits. After assembly, solder joints and supply rails should be visually inspected before powering the system. Components C_14_ and R_8_ were included solely for internal testing purposes and should not be assembled in the final device.

**Fig. 4:**
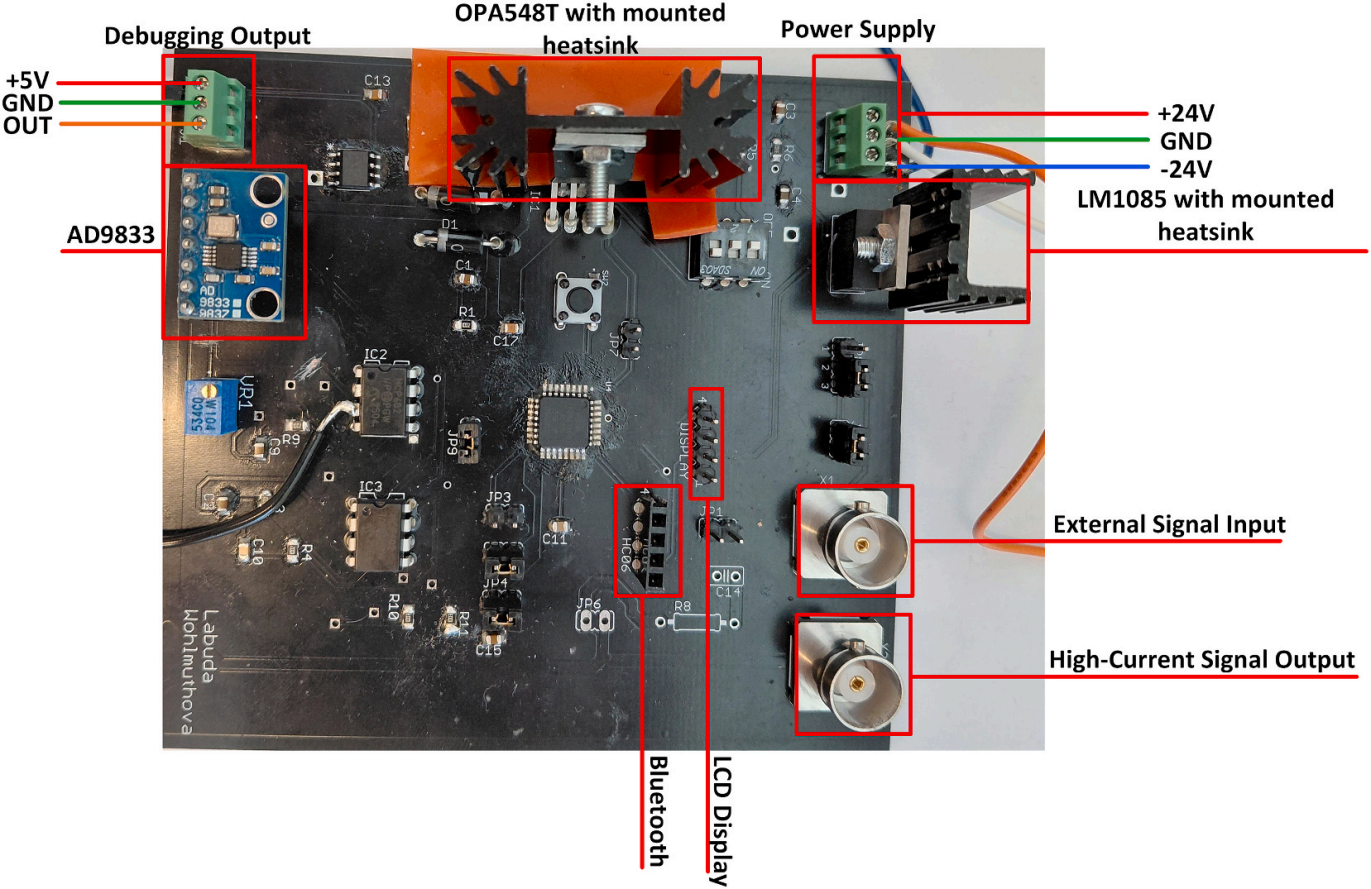
Annotated photograph of the assembled PCB with highlighted key components and connection points.

Fig. 4 illustrates the practical realization of the proposed system in the form of a fully assembled printed circuit board. The board layout highlights the placement of key functional components, interconnections, and mechanical elements required for operation. The AD9833 DDS module is implemented as a plug-in breakout board, simplifying assembly and reducing the need for fine-pitch soldering.

The power supply interface is located in the upper-right corner, providing connections for ± 24 V and ground. The high-current amplification stage, including the OPA548 operational amplifier and its associated heatsink, is centrally positioned to facilitate effective thermal dissipation. The LM1085 voltage regulator is also equipped with a heatsink to ensure stable operation of the low-voltage circuitry.

External interfaces are clearly identified, including the input connector for an external signal generator and the high-current output connector for the load. Additional connectors provide interfaces for the LCD display and Bluetooth module, enabling user interaction and wireless data transmission. A debugging header is also included for monitoring internal signals during development and testing.

The annotated layout highlights the physical organization of the system and provides guidance for assembly, wiring, and verification, thereby improving reproducibility of the proposed hardware platform.

**Fig. 5:**
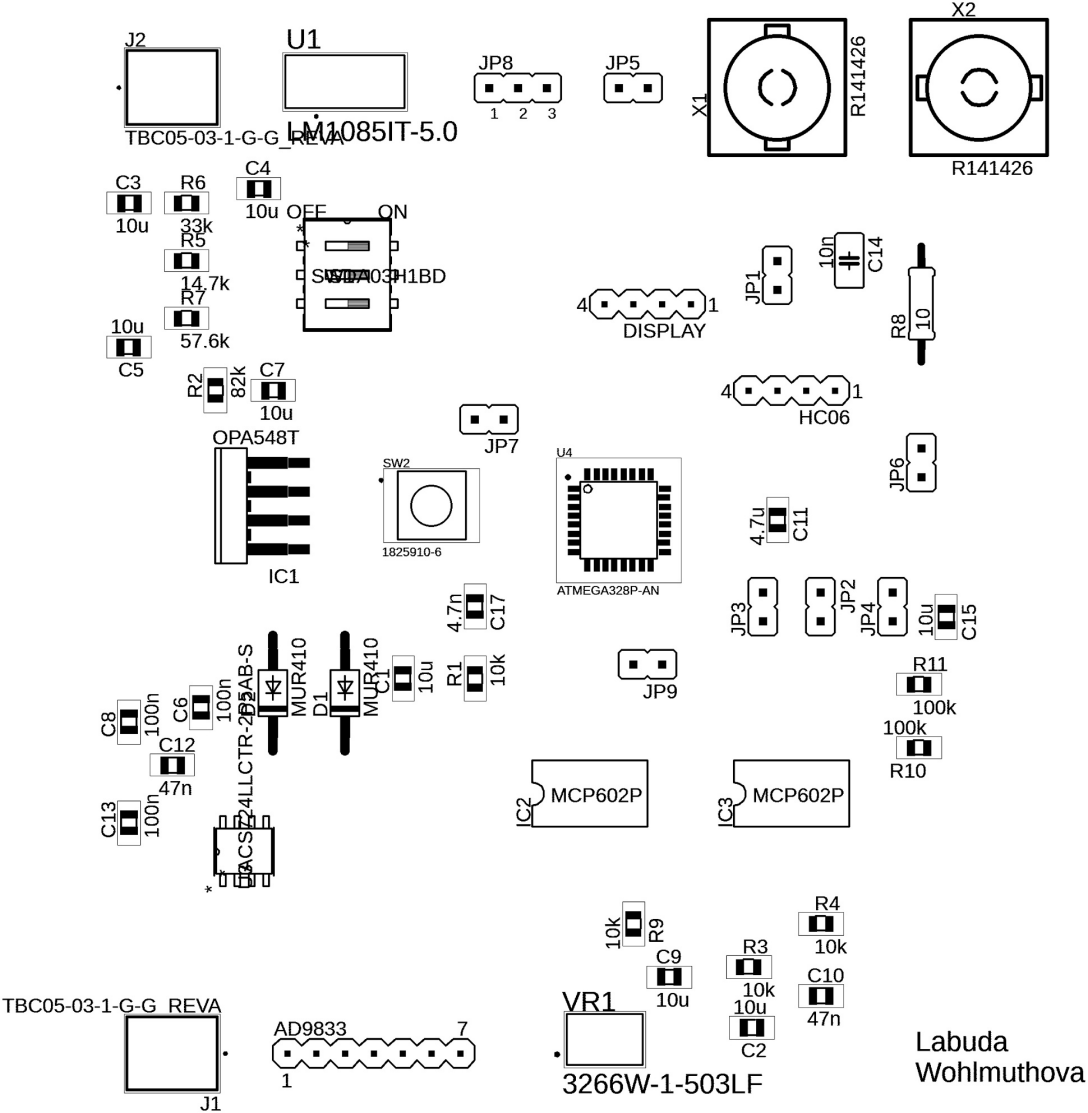
PCB Layout.

**Fig. 6:**
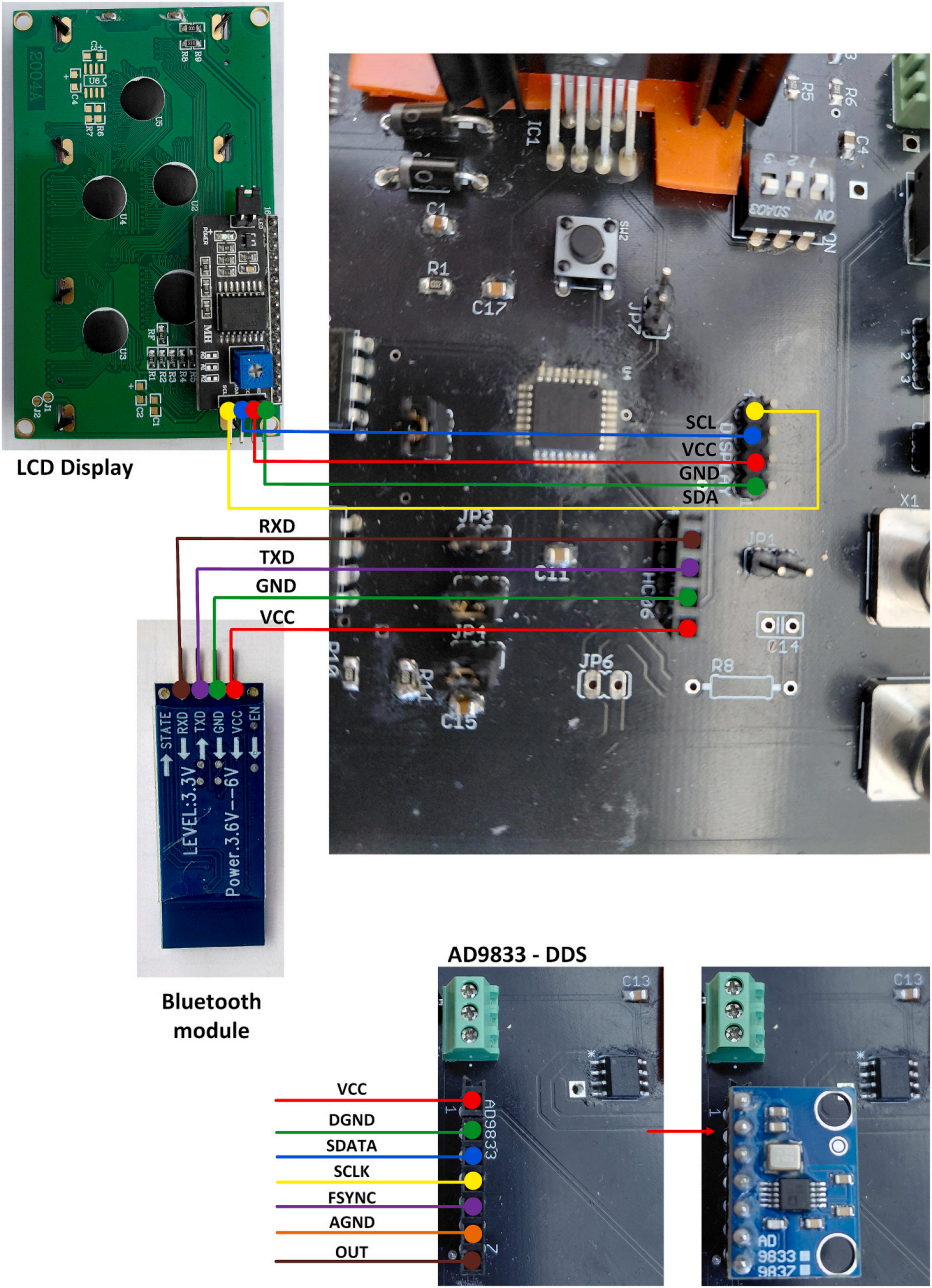
Block diagram illustrates the connections between the peripheral devices and the PCB.
• The jumper labeled “HC06” is used to connect the Bluetooth module to the designed board. The pins are ordered from 1 to 4 as follows: VCC, GND, RXD, TXD, Fig. 6.
• JP1, JP2, JP3, JP4 are jumpers used for programming the board via ISP using an Atmel ICE programmer. The pin assignments are:
  o JP1-1 (RESET)
  o JP1-2 (GND)
  o JP2-1 (MOSI)
  o JP3-1 (MISO)
  o JP4-1 (SCK)
• JP9 is the SS (Slave Select) pin for communication between the AD9833 DDS circuit and the MCU. After programming the MCU, it is also necessary to short the jumpers labeled JP2 and JP4.
• JP8 is used to select the INPUT for the designed device. The options are:
  o SINEWAVE – signal generated by the internal AD9833
  o GEN – an externally generated signal (e.g., from a signal generator) supplied to the amplifier
• JP5 is the ADC2 jumper, used to measure the voltage from the AD9833 output after initial amplification using the built-in 10-bit ADC of the ATmega328P MCU. For proper operation of the device, this jumper must be shorted.
• JP7 must never be shorted; it is used only to monitor the status of the OPA548 power amplifier.
• Screw terminal J2 is used to connect the supply voltages + 24 V, −24 V, and GND to the designed device.
• Screw terminal J1 provides the 5 V supply output, GND, and the power amplifier output.
• The power amplifier output is available on the BNC connector X2 as well as on one of the terminals of screw terminal J1.
• The external generator input can be connected to the designed device via BNC connector X1.
• SW1 is used to select the output current limit of the OPA548 operational amplifier.
• SW2 is a push-button control employed to adjust the output signal frequency within the ELF range, from 5 Hz to 300 Hz.

Description of individual jumpers, connectors and buttons (Fig. 5) and their functions:

• The jumper labeled “display” is used to connect the LCD display. The pins are ordered from 1 to 4 as follows: GND, VCC, SDA, SCL, Fig. 6.

The wiring and interconnection of peripheral modules are illustrated in Fig. 6. The figure provides a visual representation of the connections described above, including the LCD display, Bluetooth module, and AD9833 DDS module. The annotated layout highlights the required wiring between the main PCB and external modules, facilitating correct assembly and verification of the system.

The use of pre-assembled modules and clearly defined connectors simplifies integration and reduces assembly complexity. The presented wiring diagram therefore serves as a practical reference for ensuring correct interconnection of all functional blocks.

### 5.2 Heatsinks

Using heatsink on OPA548 and LM1085 (Fig. 4) is important because both devices can dissipate significant power in the form of heat during operation. If this heat is not properly removed, the temperature of the components can rise above their safe operating limits, which may lead to reduced performance, thermal shutdown, or even permanent damage.

The OPA548 is a high-current operational amplifier capable of delivering several amperes of output current. When driving heavy loads or operating with a large voltage difference between supply and output, it dissipates considerable power internally. Since power dissipation is proportional to the voltage drop across the device multiplied by the output current, high-current applications can quickly generate substantial heat. A heatsink helps transfer this heat away from the chip, maintaining a safe junction temperature and ensuring stable, reliable operation.

The LM1085 is a low-dropout (LDO) voltage regulator that also dissipates power as heat, especially when there is a significant difference between the input and output voltage. For example, stepping down from 24 V to 5 V at higher currents results in notable power loss inside the regulator. Without adequate cooling, the device may enter thermal protection mode or suffer long-term degradation. A heatsink reduces thermal resistance from the junction to ambient air, improving efficiency and preventing overheating.

### 5.3 Uploading the firmware to the MCU

Once the printed circuit board has been assembled and all components have been properly connected, the firmware must be programmed into the ATmega328P microcontroller. For this purpose, the free development environment Microchip Studio was used. The firmware file is included in the uploaded materials under the name SWD_firmware. Microchip Studio is an integrated development environment (IDE) designed for AVR and ARM microcontrollers manufactured by Microchip Technology. It enables writing, compiling, and debugging applications in C/C++, and also provides functionality for directly programming the target microcontroller using a compatible hardware programmer. In this project, the Atmel-ICE debugger/programmer was utilized. Communication between the programmer and the microcontroller was carried out via the ISP interface, which uses SPI signals. The correct pin connections required to interface the Atmel-ICE with the PCB are specified in Table 2.

**Table 2:** Pinout for connecting the Atmel-ICE Debugger to PCB [10].

**Atmel-ICE AVR ports pins**	**Target pins**
Pin 1	SCK
Pin 2	GND
Pin 3	MISO
Pin 4	5 V
Pin 6	/RESET
Pin 9	MOSI

Steps for establishing the connection between the programmer and the PCB:

1. Remove jumper JP1.
2. Connect individual programmer pins according to Table 2. and Fig. 7 (red, black, blue, green, orange, purple, yellow wires).

**Fig. 7:**
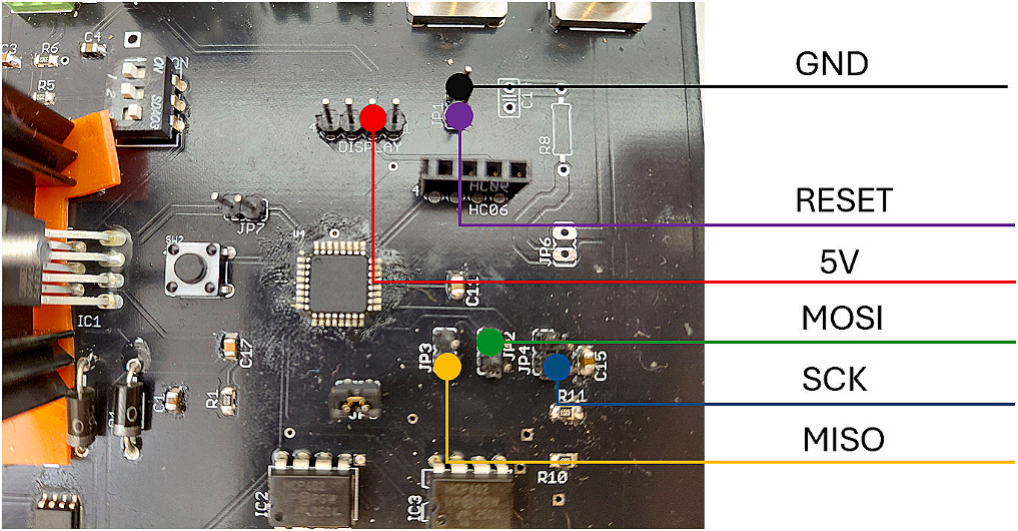
Visual representation of connection of Atmel-ICE Debugger to PCB*.*
3. Connect the Atmel-ICE programmer to the power supply.
4. Connect the power supply to control unit PCB.

Procedure for Uploading Firmware to the Control Unit:

Connect the Atmel-ICE programmer to your computer and to the target PCB, as shown in Fig. 7 and Table 2.

**Fig. 8:**
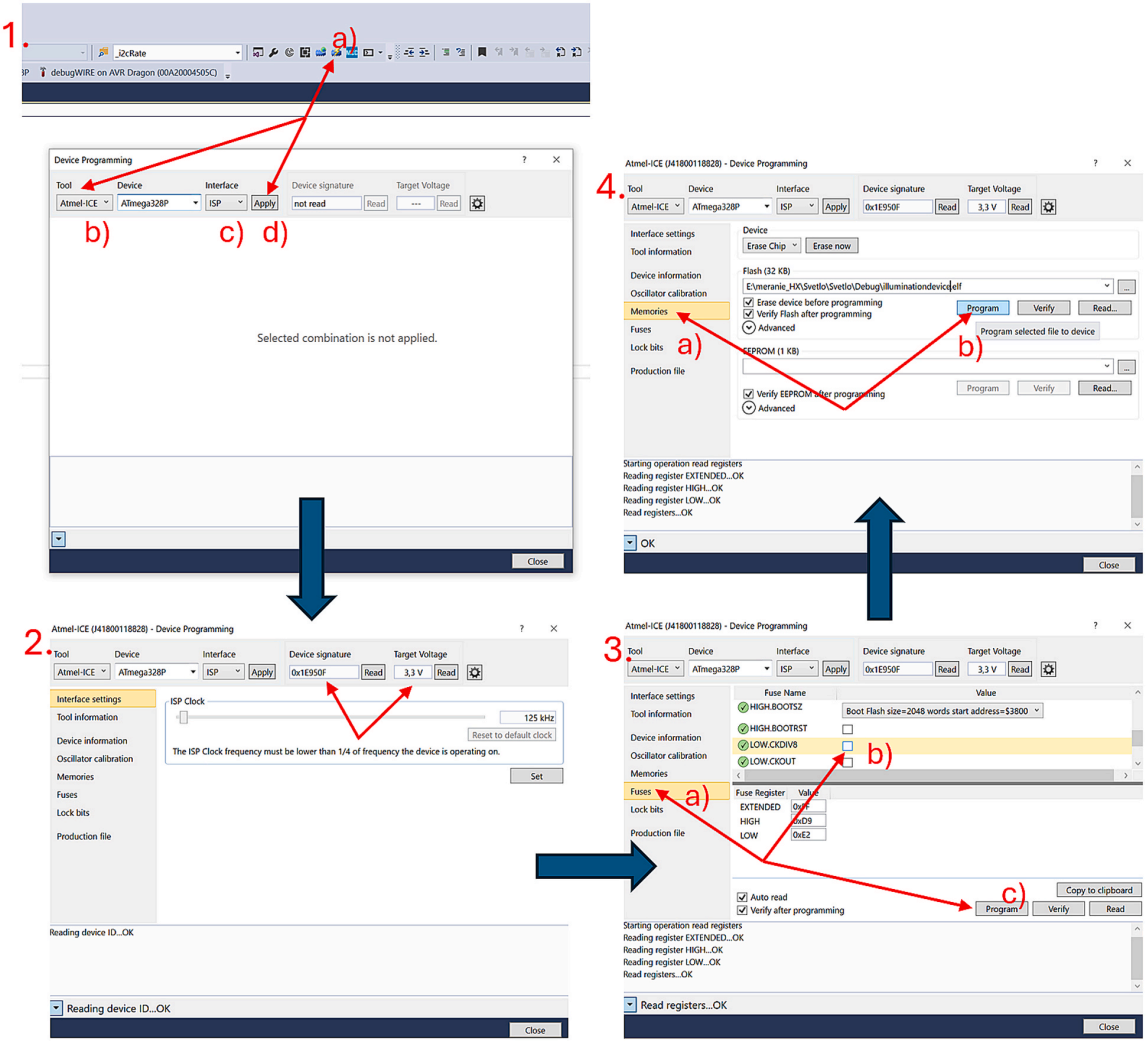
Detailed firmware upload instructions.
Check that communication with the microcontroller has been successfully established. The correct device signature (0x1E950F) and the measured target voltage (5 V) should be displayed (Fig. 8, step 2).
In the Fuses section, clear (disable) the LOW.CKDIV8 fuse to configure the MCU to operate at a clock frequency of 8 MHz. Confirm the setting by clicking Program (Fig. 8, step 3).
Next, go to the Memories tab and load the generated.hex file into the control unit by clicking Program (Fig. 8, step 4).

Launch Microchip Studio, open the relevant project, and select Device Programming. Choose Atmel-ICE as the programming tool and ISP as the interface, then press Apply (see Fig. 8, step 1).

## 6 Operation instructions

### 6.1 Stage 1: High-current operational amplifier OPA548

**Fig. 9:**
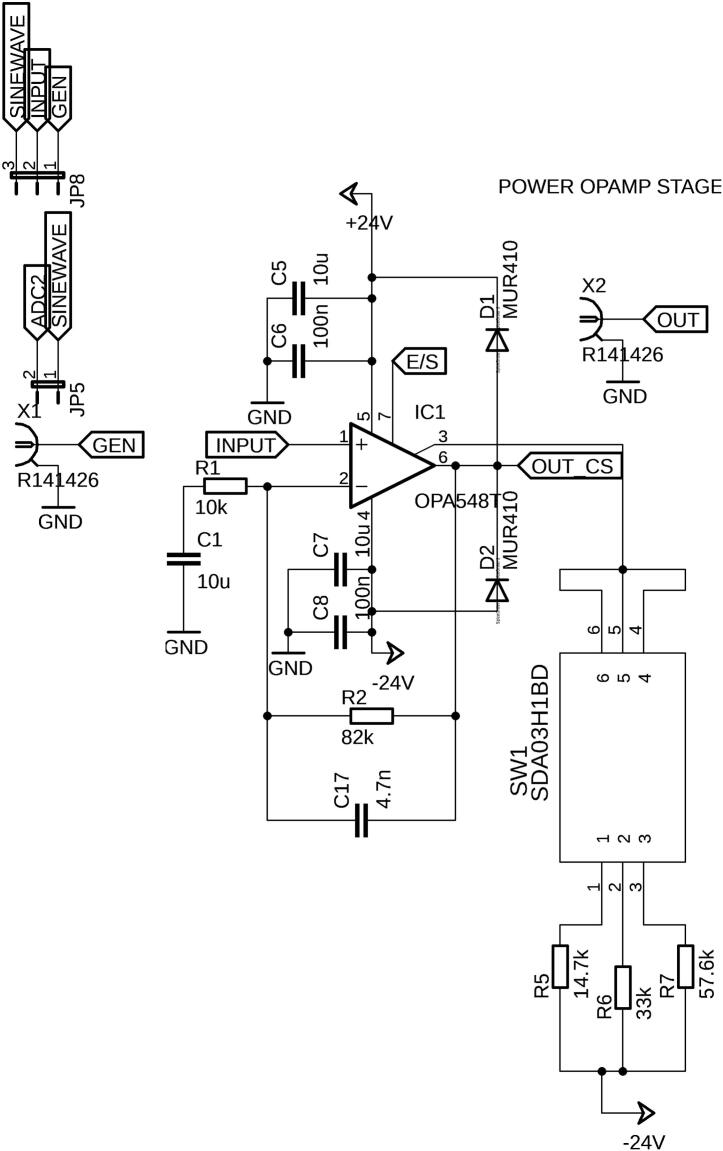
Electrical schematic of High-current operational amplifier OPA548.

The first stage described consists of the OPA548 operational amplifier [11], which forms the core of the entire designed device, as shown in Fig. 9. The operational amplifier (OA) is configured in a non-inverting topology. The input of the operational amplifier (INPUT) can be connected either to the output of an external signal generator (GEN) or to a sinusoidal signal generated by the integrated DDS circuit AD9833 (SINEWAVE). The input source is selected using jumper JP8. The amplifier is powered from a symmetrical ± 24 V supply.

The operational amplifier is preset to a gain of 9.2. The voltage gain of the non-inverting configuration can be calculated as:

(1) $$G_{OPA548}=1+\frac{R_{2}}{R_{1}}=9.2,$$

where *R*_2_ is the feedback resistor and *R*_1_ is the resistor connected to the inverting input of the operational amplifier. The selected gain can be easily modified by replacing components *R*_2_ and *C*_17_, followed by an appropriate adjustment of the cutoff frequency.

The operational amplifier also functions as a band-pass filter. The upper cutoff frequency can be calculated as:

(2) $$F_{H}=\frac{1}{2\pi R_{2}C_{17}}=412.96Hz,$$

the lower cutoff frequency can be calculated as:

(3) $$F_{L}=\frac{1}{2\pi R_{1}C_{1}}=1.59Hz.$$

The cutoff frequencies were selected to enable operation with an inductive load primarily within the ELF band.

The operational amplifier includes a dedicated pin (pin 3), labelled *I*_LIM_, which is used to implement output current limiting. According to the manufacturer’s technical documentation, the maximum output current can be adjusted by means of a resistor *R*_CL_ connected to this input. The maximum output current can be calculated using the following formula:

(4) $$I_{LIM}=\frac{\left(15000\right)Ö¼\left(4.75\right)}{13750\Omega +R_{CL}}.$$

The level of the maximum output current, shown in Fig. 9, is determined by the values of resistors *R*_5_, *R*_6_ and *R*_7_. The corresponding output current levels are summarized in Table 3.

**Table 3:** Maximum output current setting.

**Resistor Value**	14700 Ω	33000 Ω	57600 Ω
**Maximum Output Current (*I*_PEAK_)**	2.5 A	1.52 A	1 A

Ultrafast-recovery protection diodes MUR410 [12] are connected to the amplifier output. These diodes protect the operational amplifier output from inductive kickback when driving an inductive load.

### 6.2 Stage 2: Programmable waveform generator AD9833

The second stage consists of an internal sinusoidal signal generator. In the proposed device, a DDS integrated circuit AD9833 is employed [13]. The DDS enables straightforward generation of a sinusoidal signal in the frequency range from 0 Hz to 12.5 MHz with an output amplitude of 0.60 V_PP_. The output signal of the AD9833 includes a DC offset of 0.325 V.

**Fig. 10:**
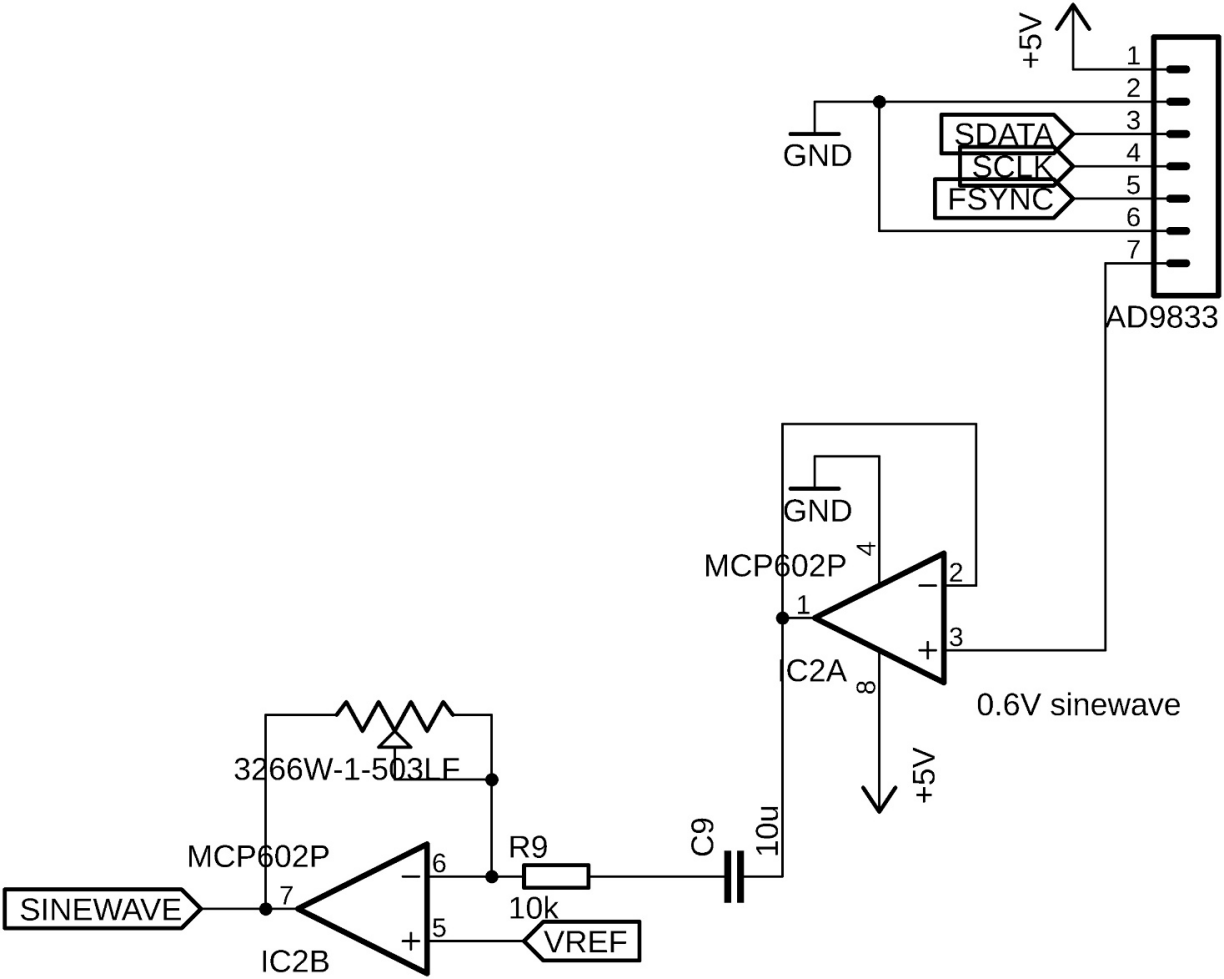
Electrical schematic of Programmable waveform generator AD9833.

The output of the AD9833 is connected to a buffer implemented using an MCP602 operational amplifier [14], as shown in Fig. 10, to provide impedance isolation between the signal source and subsequent stages of the system. The buffered signal is then fed into an active high-pass filter that also operates as an inverting amplifier, realized using an MCP602 operational amplifier. The gain and cutoff frequency of the filter can be calculated using the following formulas:

(5) $$F_{C}=\frac{1}{2\pi R_{9}C_{9}}=1,59Hz,$$

(6) $$G_{AD9833}=-\frac{R_{9}}{R_{POT}},$$

where $R_{POT}$ represents the resistance of a multi-turn potentiometer with a maximum resistance value of 50 kΩ. The maximum achievable gain, assuming a DC offset of 2.5 V, is − 3.5. Under these conditions, the amplified sinusoidal output signal reaches a peak-to-peak amplitude of 2.1 V_PP_.

The overall maximum gain of the internally generated sinusoidal signal at the output of the OPA548 stage is given by:

(7) $${G=G}_{AD9833\;}Ö¼G_{OPA548}=9.2Ö¼\left(-3.5\right)=-32.2.$$

After amplification by the OPA548 operational amplifier, the output voltage reaches 19.32 V_PP_. Communication between the DDS circuit and the microcontroller is implemented via the Serial Peripheral Interface (SPI).

### 6.3 Stage 3: Hall-effect current sensor ACS724LLCTR-2P5AB-S

The sensor has a measurable range of ± 2.5 A. The sensor provides a voltage output proportional to the instantaneous current value, with a sensitivity of 800 mV/A [15]. The output voltage exhibits a DC offset of 2.5 V. The ACS724 incorporates an internal passive RC filter, whose cutoff frequency is configured to correspond to the ELF band, according to the following formula:

(8) $$F_{C}=\frac{1}{2\pi \cdot 1800\Omega {\cdot C}_{12}}=1881.26Hz,$$

**Fig. 11:**
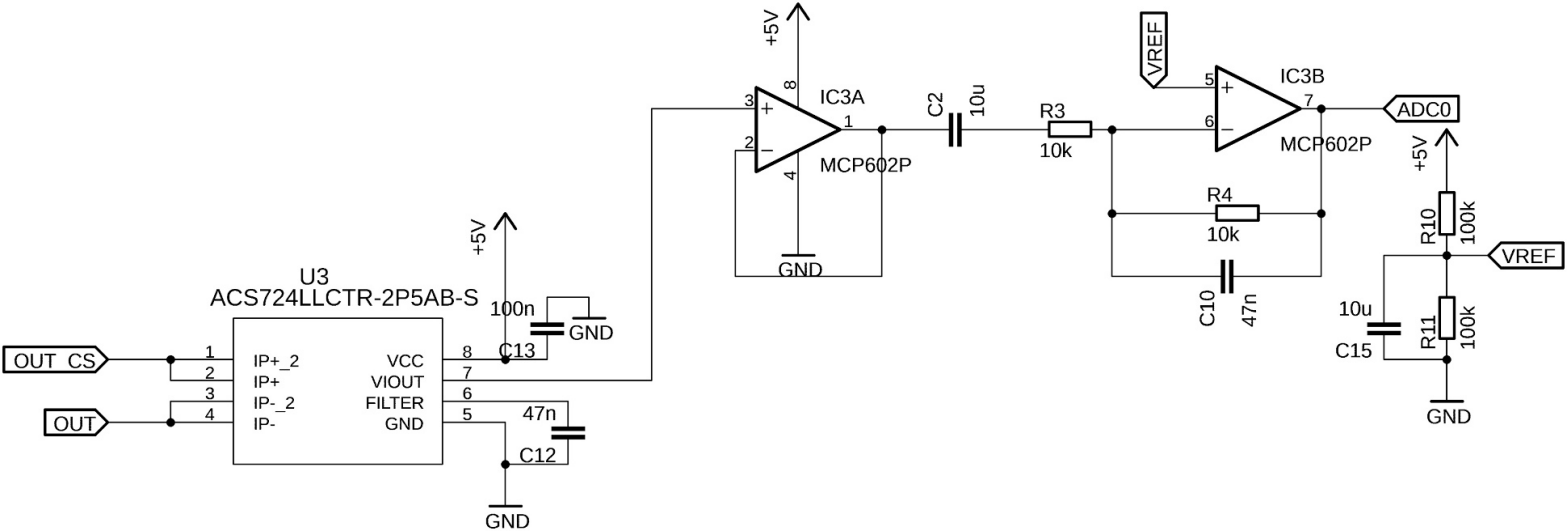
Electrical schematic of Hall-effect current sensor ACS724.

The cutoff frequency *F*_c_ is intentionally shifted to prevent distortion of the measured current values through the load, as recommended by the manufacturer. At the same time, the filter effectively suppresses high-frequency interference. The sensor output is fed into a buffer (Fig. 11) and subsequently into an operational amplifier configured as a band-pass filter with a gain of − 1.

The cutoff frequencies of the BP filter can be calculated as:

(9) $$F_{H}=\frac{1}{2\pi R_{4}C_{10}}=338.63Hz,$$

(10) $$F_{L}=\frac{1}{2\pi R_{3}C_{2}}=1.59Hz.$$

With the selected frequencies, it is possible to effectively measure the output current of the OPA548 within a frequency range from 5 Hz to 300 Hz and a current range of 0–2.5 A_PP_. At the same time, the requirement for compliance with the ELF band is maintained.

### 6.4 Stage 4: MCU and Bluetooth communication

**Fig. 12:**
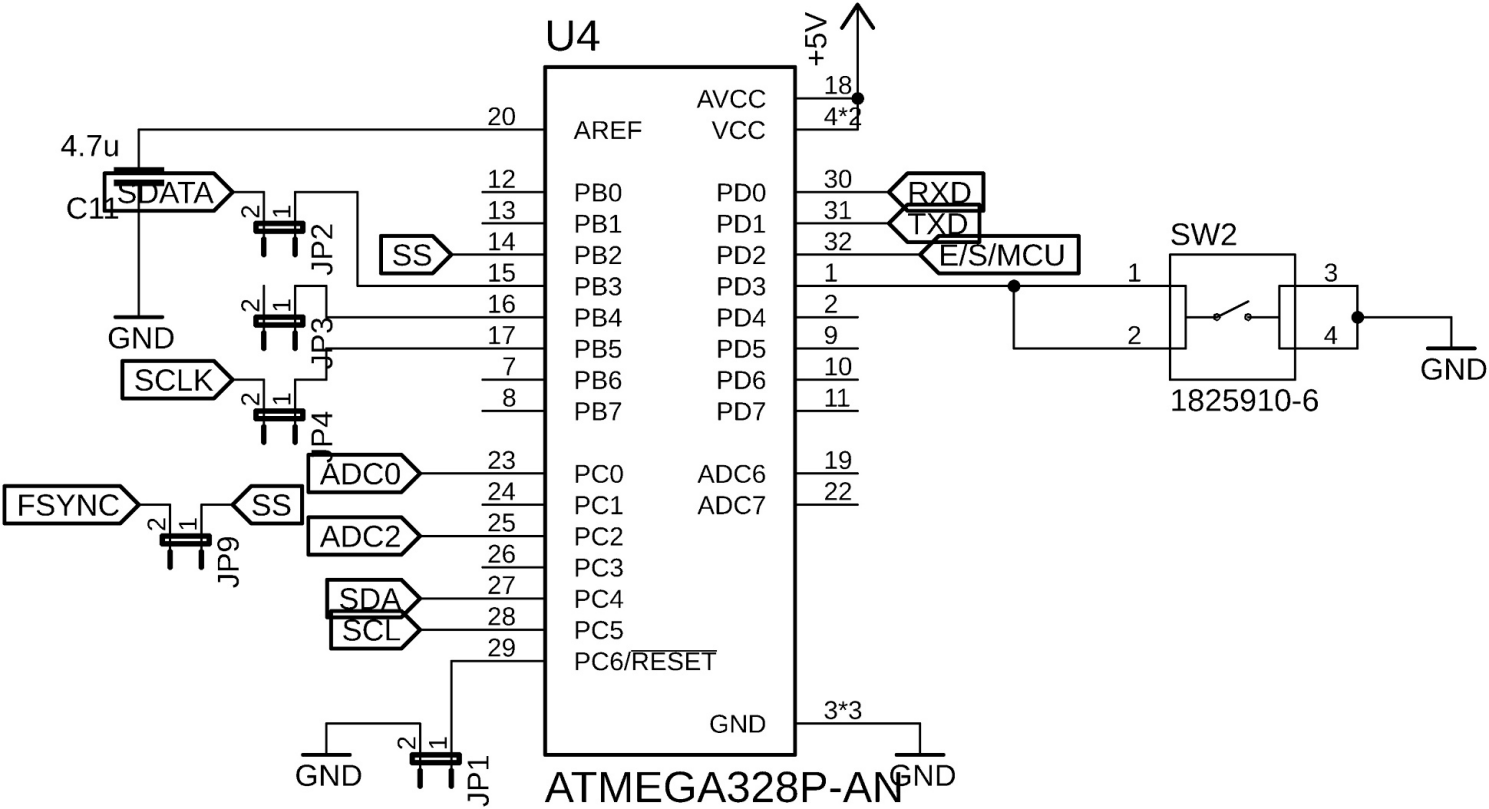
Electrical schematic of MCU circuit.

Stage 4 is represented by a microcontroller designated ATmega328P [16], Fig. 12. The MCU provides control of the AD9833 DDS circuit, enabling selection of the desired frequency component within the ELF range. At the same time, the MCU is used to acquire the output signal from the ACS724 circuit in order to calculate the RMS value of the current to the load at the output of the OPA548 amplifier.

The output of the AD9833 generator is also connected to the MCU, allowing straightforward determination of the amplitude of the generated sinusoidal signal. The RMS values of the load current and the generator voltage are displayed on an LCD module, which communicates with the MCU via the I^2^C communication interface. In addition, a Bluetooth HC-06 [17] module is connected to the MCU to enable wireless transmission of the measured data to a computer. The push button (SW2) is used to adjust the frequency in 5 Hz increments within the ELF range, from 5 Hz up to 300 Hz.

### 6.5 Firmware operation

The firmware for the ATmega328P microcontroller is written in C programming language and is responsible for signal generation control, data acquisition, user interface management, and communication with external devices. After system start-up, the microcontroller initializes the SPI, ADC, UART, Timer1, and I^2^C interfaces. The SPI interface is used for communication with the AD9833 DDS generator, the ADC for sampling analog signals, the UART for Bluetooth communication, and Timer1 for periodic data acquisition.

**Fig. 13:**
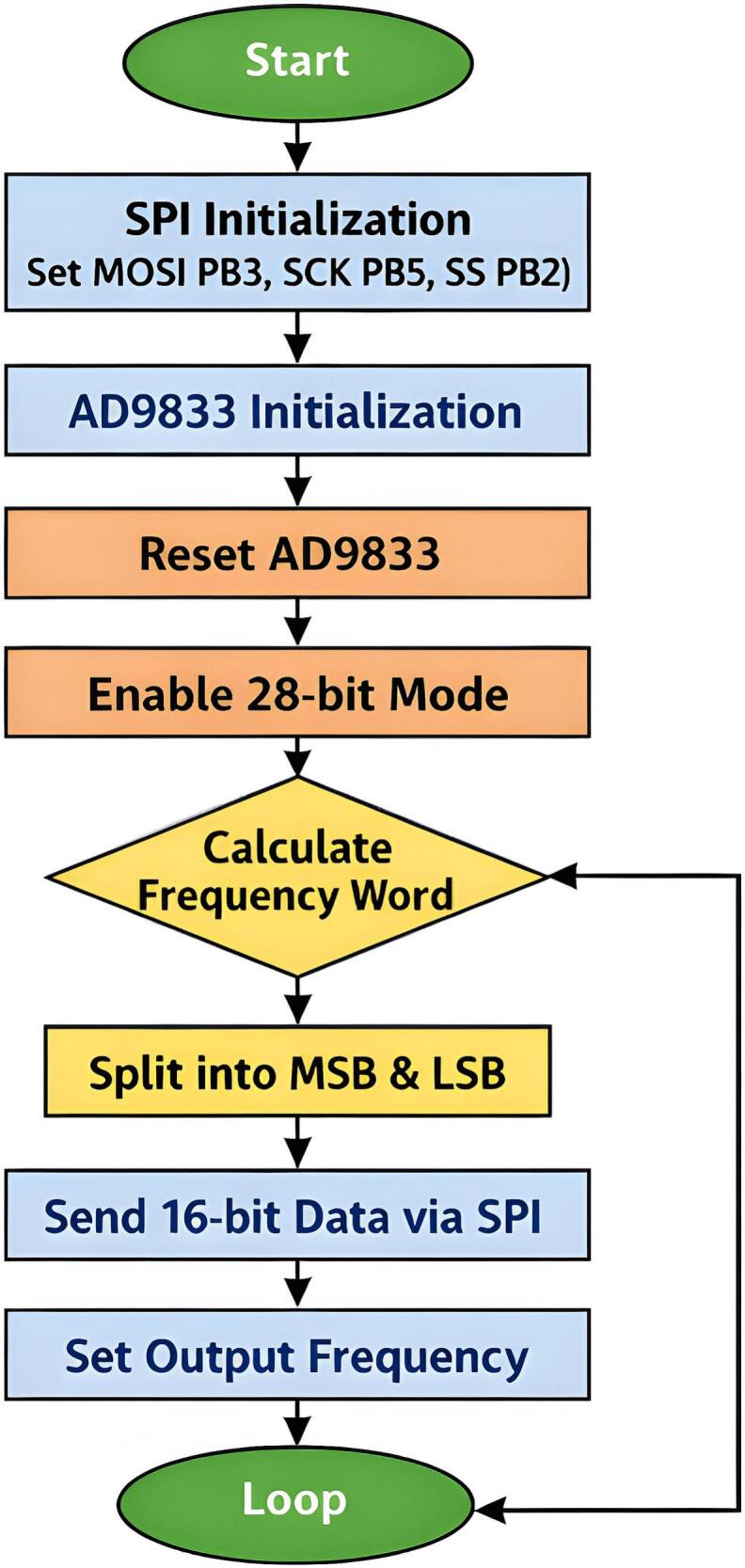
Managing frequency change at AD9833 output.

The output frequency is generated using the AD9833 DDS circuit, which is controlled via the SPI interface. The flowchart shown in Fig. 13 illustrates the communication and control sequence between the ATmega328P microcontroller and the AD9833. After initialization, the SPI peripheral is configured by setting the MOSI (PB3), SCK (PB5), and chip-select/FSYNC (PB2) pins, and enabling master mode operation. The AD9833 is then reset via its control register to stop waveform generation and clear internal frequency and phase accumulators, ensuring deterministic behaviour.

The device is subsequently configured to operate in 28-bit frequency mode, which provides high frequency resolution. The required frequency tuning word is calculated by the microcontroller based on the desired output frequency and the reference clock of the AD9833. This 28-bit value is divided into two 14-bit segments, which are transmitted sequentially via the SPI bus. After transmission, the DDS output frequency is updated and waveform generation resumes. During operation, the frequency can be modified dynamically, and the tuning word is recalculated and updated accordingly.

Analog measurement is performed using two ADC channels. One channel corresponds to the conditioned current-sensor output, and the second monitors the generated signal. Sampling is performed periodically using a Timer1 interrupt. At each interrupt, both ADC channels are sampled and accumulated. The firmware stores the sum of samples and the sum of squared samples over a block of 100 measurements.

After collecting 100 samples, the RMS value is calculated using a DC-offset-compensated algorithm based on accumulated sums and squared sums. The resulting RMS values are converted from ADC counts to millivolts using the reference voltage and further scaled to represent the output voltage and load current according to the analog front-end characteristics.

The measured values are displayed on a 20 × 4 LCD using the I^2^C interface. The display shows the RMS voltage, RMS current, and the selected output frequency. The output frequency can be adjusted by a push button, which increments the frequency in 5 Hz steps within the range of 5 Hz to 300 Hz.

In addition to local display, the firmware supports wireless data transmission via a Bluetooth module. When a start command is received over UART, raw ADC samples are transmitted continuously, enabling real-time visualization of voltage and current waveforms in the MATLAB application. Transmission can be stopped using a corresponding command.

Overall, the firmware provides coordinated control of signal generation, periodic data acquisition, real-time measurement processing, and user interaction, ensuring stable and reproducible operation of the device.

### 6.6 MATLAB application operation

**Fig. 14:**
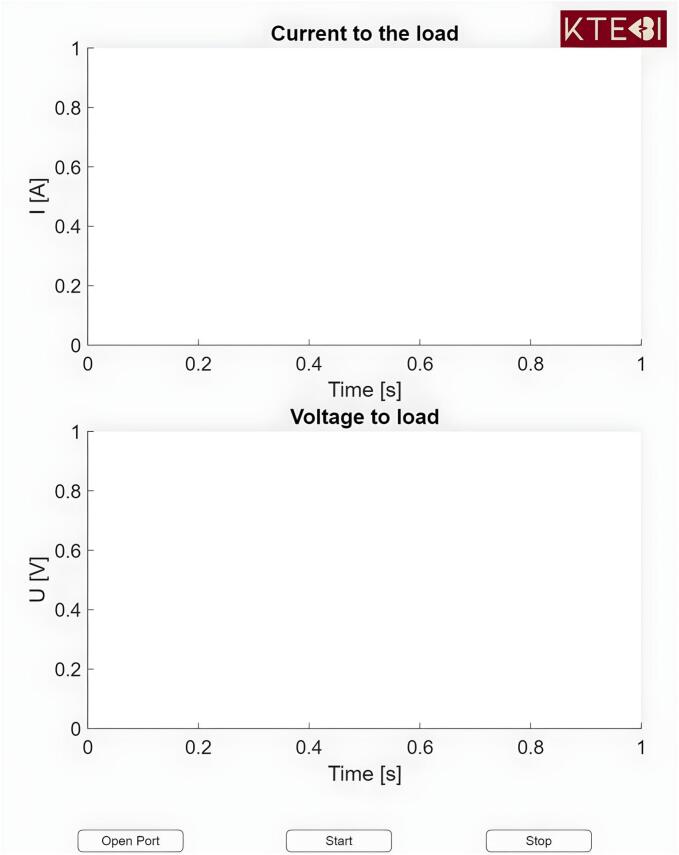
MATLAB Application GUI.

To enable visualization of the load voltage and current waveforms, an auxiliary application was developed in MATLAB using App Designer (Fig. 14). The application communicates with the device through a Bluetooth serial interface configured at 38 400 baud and provides real-time display of the acquired signals in two separate plots: one for load current and one for load voltage.

After start-up, the application initializes the graphical interface and enables only the serial-port opening function, while data acquisition controls remain disabled until communication is established. Once the communication port is opened, animated plotting objects are created for both channels, allowing continuous real-time waveform updates.

Data acquisition is initiated by the user through the “Start” button, which sends the command character ‘S’ to the microcontroller to enable streaming of measurement data. During operation, the application continuously monitors the serial buffer and reads incoming data as 16-bit samples. The samples are separated into current and voltage channels, stored in internal arrays, and simultaneously plotted in real time.

The visualization is implemented using a moving 10 s time window. As new data are received, they are appended to the plotted waveforms, and once the displayed interval is filled, the plots are cleared and the time axis is shifted to the next segment. This approach enables continuous monitoring while maintaining clear visualization.

The sampling frequency used for data acquisition is configurable and can be adjusted according to the measurement requirements. In the default MATLAB configuration, a sampling frequency of 500 Hz is used, which satisfies the Nyquist sampling criterion for signal frequencies up to 250 Hz. Therefore, this default setting is suitable for waveform visualization within the 5–250 Hz range. For operation at higher frequencies, including 300 Hz, the sampling rate can be increased in the microcontroller firmware to ensure that the Nyquist sampling criterion remains satisfied.

Data acquisition is terminated using the “Stop” button, which sends the command character ‘X’ to the microcontroller to disable data transmission. The acquired current and voltage data are then exported to the MATLAB workspace for further analysis, enabling additional processing such as waveform inspection, RMS evaluation, or harmonic distortion analysis.

Overall, the MATLAB application serves as a diagnostic and visualization tool that complements the hardware platform by enabling both real-time monitoring and post-processing of the measured signals.

### 6.7 Stage 5: Power supply

**Fig. 15:**
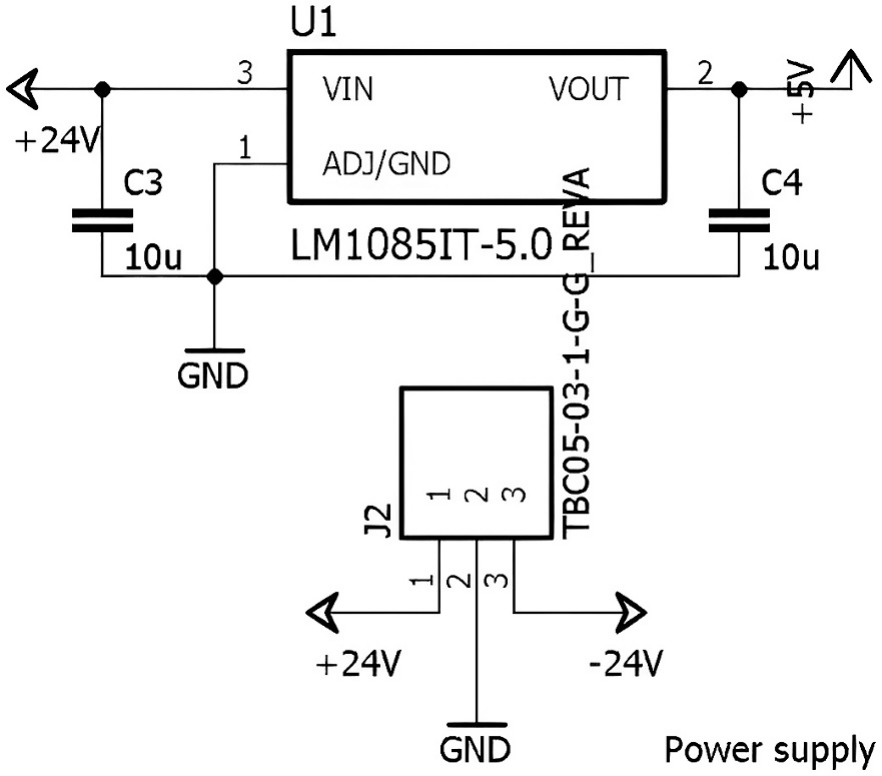
Electrical schematic of the voltage regulation circuit.

The system is powered by two IDEC PS3V-100AF24C + 24 V, 4.5 A [18] switched-mode power supplies. The supplies are connected in such a way that their output voltages form a ± 24 V supply rail. The integrated circuits used in the device, except for the OPA548, require a + 5 V supply voltage, therefore, a LDO fixed voltage regulator LM1085IT-5.0 [19] was used in the design, Fig. 15.

## 7 Validation and characterization

The proposed device was evaluated from three main perspectives. First, the stability of the system was tested during power-on and power-off conditions while supplying an inductive load. Subsequently, the generated sinusoidal signal was analysed in terms of its frequency components and spectral purity at selected output frequencies. Finally, the reliability of generating a sinusoidal signal with the required amplitude was verified, and the current sensor was tested for measuring the load current under both resistive (R) and resistive–inductive (RL) load conditions.

**Fig. 16:**
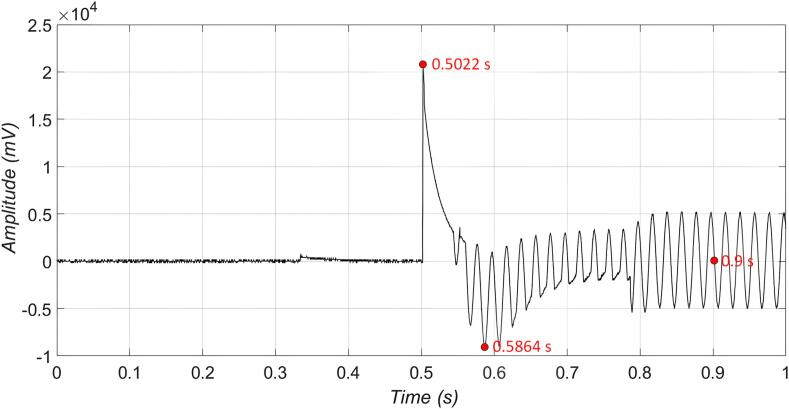
Turn-on voltage transient phenomenon to RL Load, recorded by oscilloscope OWON VDS1022.

Fig. 16 shows the voltage transient response of an RL load during switching-on. From the beginning up to approximately 0.5 s, the voltage remains close to zero, with only small fluctuations visible. This indicates that the load is not yet energized and the system is in a steady state.

At around 0.502 s, a very sharp positive voltage spike occurs. The voltage suddenly rises to approximately 20800 mV. This large spike is characteristic of an inductive load, as the inductor opposes the sudden change in current when the circuit is switched on. The voltage level stays below 24 V because the MUR410 protection diodes are used at the OPA548 output.

Immediately after this peak, the voltage rapidly decreases in an exponential-like manner. Shortly afterward, a significant negative overshoot appears, reaching about –9200 mV at approximately 0.586 s. This behaviour indicates oscillatory effects in the circuit, likely caused by the interaction between the inductance of the load and parasitic capacitances.

Following the negative overshoot, the waveform exhibits damped oscillations. The amplitude of these oscillations gradually changes and then settles into a more regular periodic pattern. Toward the end of the time interval, around 0.9 s, the oscillations stabilize with an amplitude of approximately ± 5000 mV.

Overall, Fig. 16 illustrates a high-voltage transient at the moment of switching, followed by overshoot and oscillatory behaviour before reaching a more stable steady-state condition. In the case of disconnecting the RL load, no significant voltage spikes or back-EMF transients were observed.

**Fig. 17:**
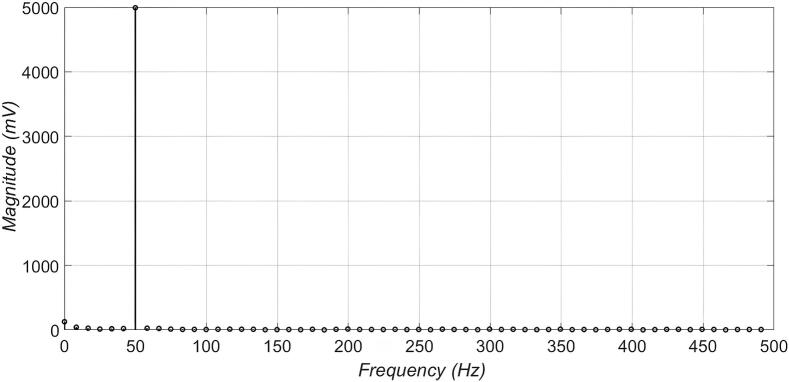
FFT of 50 Hz sinewave generated by proposed device.

Fig. 17 shows the Fast Fourier Transform (FFT) of a stabilized 50 Hz sine wave, with frequency on the horizontal axis and magnitude (in volts) on the vertical axis.

A single dominant peak appears at 50 Hz, with a magnitude of approximately 5000 mV. This indicates that the signal is primarily composed of a fundamental frequency component at 50 Hz. The peak is very sharp and well-defined, which confirms that the waveform is stable and periodic.

Apart from the main component at 50 Hz, there are no significant peaks at other frequencies. The remaining frequency components across the spectrum from 0 to 500 Hz have very small magnitudes, close to zero. This shows that the signal contains negligible harmonic distortion and very little noise.

Overall, the FFT confirms that the waveform is a clean and stabilized sinusoidal signal at 50 Hz, with no noticeable higher-order harmonics or unwanted frequency components.

The total harmonic distortion (THD) of the generated sinusoidal signal was evaluated using MATLAB based on sampled output data. The measured THD values were − 53.5 dB (0.21%) at 50 Hz, −56.9 dB (0.14%) at 100 Hz, −56.3 dB (0.15%) at 150 Hz, −58.8 dB (0.11%) at 200 Hz, and − 61.2 dB (0.09%) at 300 Hz. The results demonstrate low harmonic distortion throughout the intended operating range, including the maximum specified frequency of 300 Hz. The low THD values are supported by the DDS-based signal generation architecture and by the analog signal-conditioning stages, which suppress unwanted frequency components and high-frequency noise.

### 7.1 Stage 1: Resistive (R) load verification

**Fig. 18:**
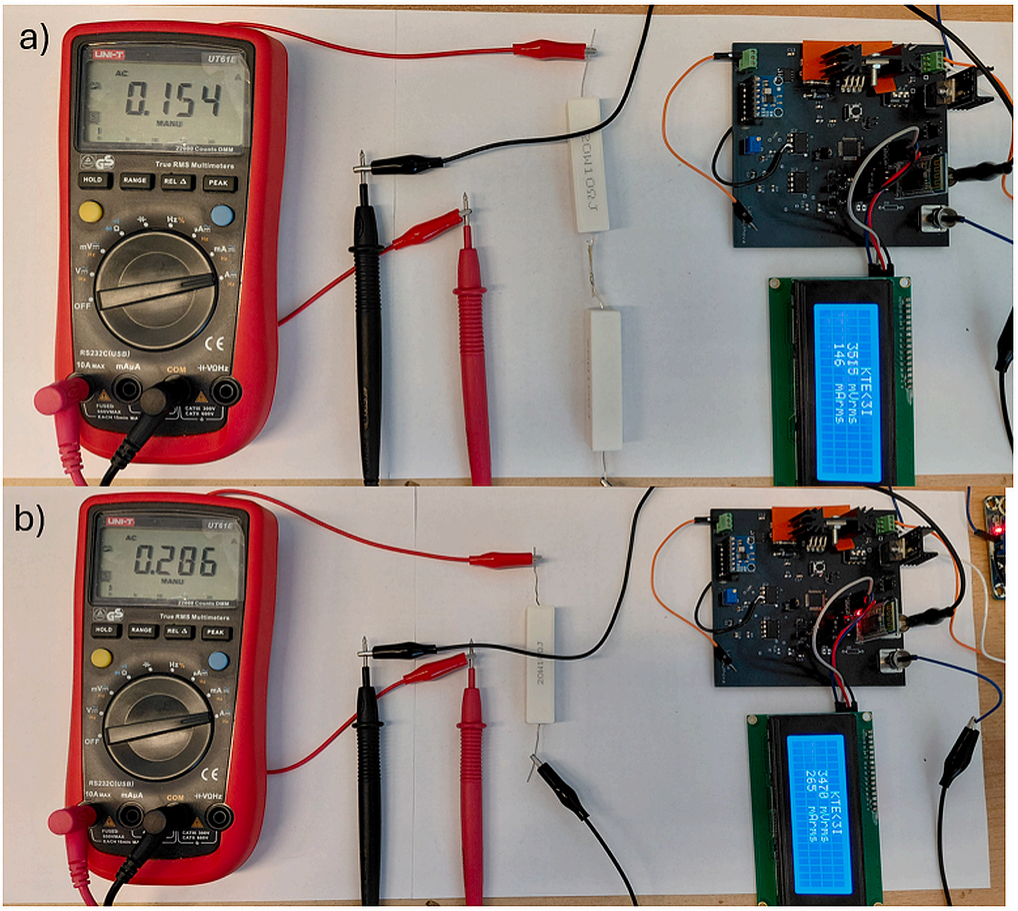
Resistive Load Measurement Setup.

The device was tested with a resistive load of 20 Ω Fig. 18 (a). According to the device readings, the RMS voltage across the load was 3515 mV and the RMS current through the load was 146 mA, Fig. 18 (a).

For verification purposes, the load voltage was independently measured using a digital oscilloscope OWON VDS1022 [20], which indicated an RMS value of 3535 mV. The deviation between the voltage displayed by the proposed device and the value measured by the oscilloscope remained at < 1 %, and similar deviations < 1 % were observed for other voltage levels.

The RMS current through the load was additionally verified using a digital multimeter UNI-T UT 61E TrueRMS [21], yielding a measured value of 154 mA. The current sensing deviation of the ACS724-based measurement circuit was around 6 % under these conditions. Furthermore, at higher load current levels above 500 mA RMS, the accuracy of current measurement improves due to the resolution of the ADC (10 bits only) and the sensitivity of the ACS724 sensor (800 mV/A). For improved accuracy, the use of an external 16-bit ADC is recommended.

The same test was also performed with a 10 Ω resistive load (Fig. 18(b)), and the measurement results were recorded in Table 4, in the same manner as in the first case.

**Table 4:** Comparison between measured and calculated RMS current to various loads. The effective value of the resistive load is increased due to the additional resistance of the measurement leads.

**Measurement Setup**	**Calculated**	**Multimeter**	**ACS724**	**Error (Calculated vs. ACS724) (%)**
R (22.6 Ω)	156.41 mA RMS	154 mA RMS	146 mA RMS	6.66 %
R (12.6 Ω)	280.55 mA RMS	286 mA RMS	265 mA RMS	5.54 %
RL (20 Ω)	176.75 mA RMS	181 mA RMS	169 mA RMS	4.38 %

### 7.2 Stage 2: Verification of an Inductive–Resistive (RL) load

**Fig. 19:**
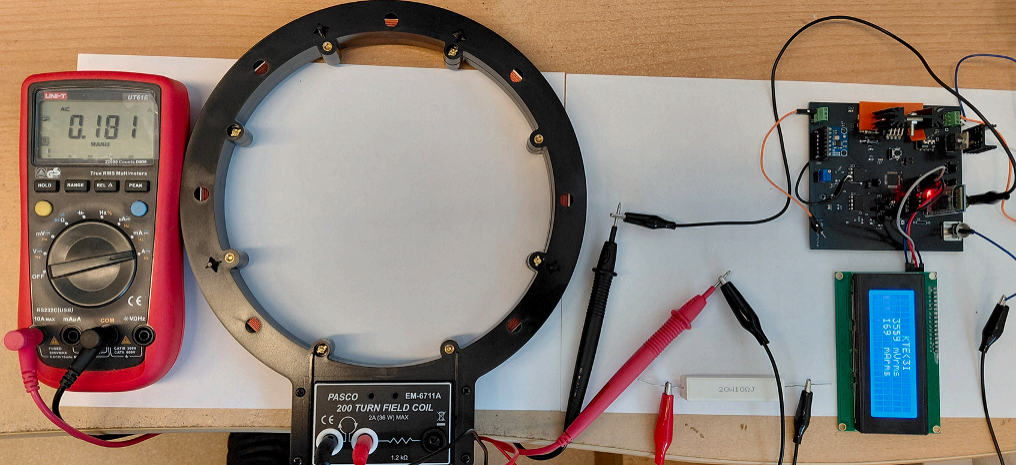
Inductive-Resistive Load Measurement Setup.

During the testing, a Helmholtz coil PASCO EM-6711A (N = 200, I_max_ = 2 A) [22] was used, Fig. 19. It was connected in series with a 10-ohm resistor. The inductance of the coil is 16.55 mH, and the winding resistance is 7.8 Ω.

The impedance of the coil (powered by 50 Hz sinusoidal voltage source) can be determined as follows:

(11) $$Z=\;\sqrt{{7,8}^{2}+{\left(2\cdot \pi \cdot 0,01655\cdot 50\right)}^{2}}=9,734\;\Omega .$$

The input voltage is the same as in the previous case, i.e., 10 V_PP_ or 3535 mV RMS. This was verified in the same manner as before using a digital oscilloscope. The voltage displayed on the display is 3559 mV RMS. The load current measured by the ACS724 sensor is 169 mA RMS. The RMS current value measured with a multimeter is 181 mA RMS. This represents a measurement deviation of less than 5 % for the current flowing through the RL load. The measurement is also influenced by the resistance of the connecting wires used to connect the RL load and the multimeter in series with the load.

**Fig. 20:**
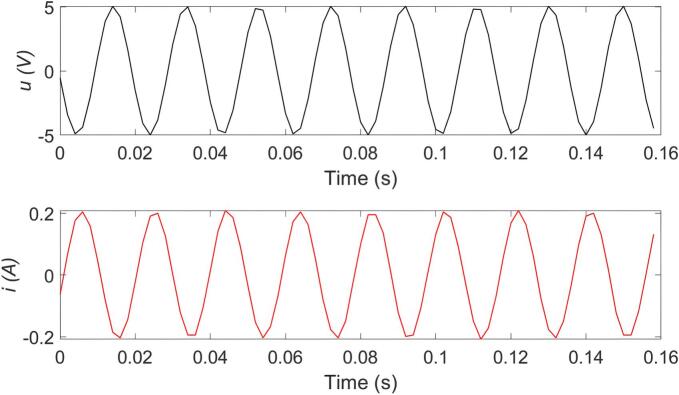
Output voltage and current to the RL Load (20 Ω) – obtained via Bluetooth & MATLAB application.

The measured voltage and current waveforms obtained using the developed MATLAB application are shown in Fig. 20 for the connected RL load. The slight distortion of the displayed sine wave is caused by the limited number of samples per period used during this particular measurement. For the 50 Hz signal shown in Fig. 20, a sampling frequency of 250 Hz was used, resulting in five samples per period. Consequently, the waveform appears as a piecewise approximation of the sinusoid. The sampling frequency is configurable and can be adjusted according to the operating frequency and measurement requirements. Furthermore, the FFT of the signal captured using the oscilloscope (Fig. 17) confirms that the generated output contains only the selected frequency component, in this case a 50 Hz sinusoidal signal.

Despite minor deviations in the measured load current values, the current remains stable even during prolonged operation. The stability of the device was verified under RL load conditions over a continuous measurement period of one hour.

The measured deviation of the current sensing circuit based on the ACS724 Hall-effect sensor was observed to be in the range of 4–6% when compared to reference measurements obtained using a calibrated multimeter (Table 4). Several factors contribute to this deviation.

First, the ACS724 sensor exhibits inherent accuracy limitations, including offset error and sensitivity tolerance specified by the manufacturer. These non-idealities introduce systematic errors, particularly at lower current levels where the relative impact of offset becomes more significant.

Second, the resolution of the analog-to-digital converter (ADC) in the ATmega328P microcontroller (10-bit) limits the precision of digitized measurements. Given the sensor sensitivity of 800 mV/A and a 5 V reference voltage, the effective current resolution is approximately 6 mA per ADC step, which limits measurement precision at low currents. This quantization effect becomes more pronounced at low current amplitudes, contributing to the observed deviation.

Third, measurement accuracy is influenced by electrical noise and interference in the analog front-end, including power supply ripple, electromagnetic coupling from the high-current amplifier stage, and switching noise from digital components. Although band-pass filtering is implemented, residual noise can still affect the measured current signal.

Additionally, parasitic resistances in connecting wires and measurement leads introduce small voltage drops, which affect the reference measurement obtained by the multimeter, leading to discrepancies between calculated and measured values.

It was also observed that measurement accuracy improves at higher current levels (above approximately 500 mA RMS), where the signal-to-noise ratio is higher and the relative influence of ADC quantization and sensor offset is reduced.

Overall, the achieved measurement accuracy is sufficient for the intended applications in laboratory experimentation, educational use, and inductive load characterization. For applications requiring higher precision, the use of an external higher-resolution ADC (e.g., 16-bit) or digital filtering techniques could further improve measurement performance.

The design prioritizes low cost, compact integration, and ease of reproducibility over high-end measurement accuracy, resulting in moderate current sensing deviations while maintaining sufficient performance for ELF applications.

### 7.3 Thermal characterization

**Fig. 21:**
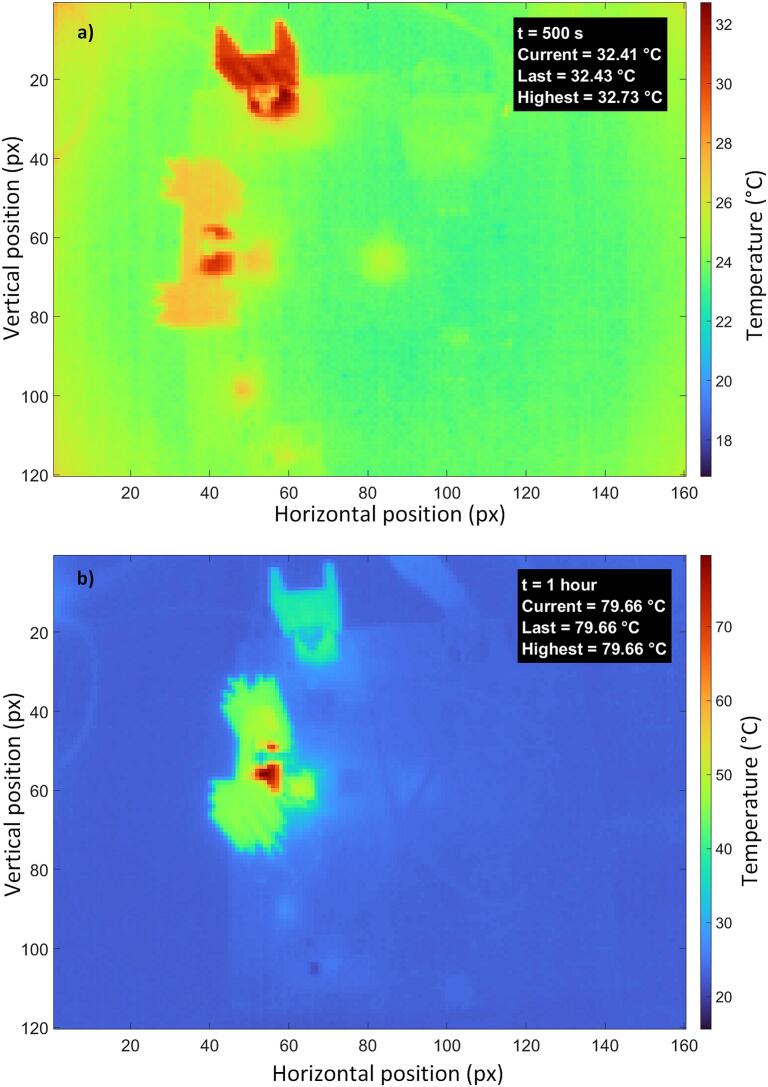
Thermal images of the system during operation: (a) no-load condition after 500 s; (b) operation under RL load at 1 A_PP_ after 1 h.

The thermal behaviour of the system was evaluated during continuous operation under RL load conditions. The thermal distribution of the system under no-load and load conditions is shown in Fig. 21, where Fig. 21(a) corresponds to the no-load condition after 500 s and Fig. 21(b) shows the temperature distribution after one hour of operation at a load current of approximately 1 A_PP_. During this test, the measured heatsink temperature of the OPA548 amplifier reached 79.66 °C, while under no-load conditions the temperature was 32.73 °C after the same duration. No thermal shutdown or observable performance degradation was detected during operation. These results indicate that the applied heatsinking provides adequate thermal management for the tested operating conditions. However, operation at higher load currents or prolonged use near the maximum output level may require enhanced thermal management or active cooling.

### 7.4 Conclusion

The presented device demonstrates a practical and flexible solution for the generation and amplification of sinusoidal excitation signals intended for driving inductive loads. By combining a programmable DDS-based waveform generator with a high-current operational amplifier, the system enables precise control of excitation frequency and amplitude while maintaining stable operation under inductive loading conditions. The ability to operate either with an internally generated sinusoidal signal or with an external laboratory function generator enhances the adaptability of the platform for a wide range of experimental and testing scenarios.

The use of the AD9833 DDS module provides stable sinusoidal waveforms, while the buffering and filtering stages ensure proper signal conditioning and impedance isolation between functional blocks. The OPA548 power operational amplifier proved effective for operation in the ELF range, offering sufficient current drive capability, integrated current limiting, and reliable protection against overload and inductive kickback. The inclusion of fast-recovery protection diodes further improves the robustness of the output stage when driving inductive elements.

Integrated real-time current monitoring using a Hall-effect sensor enables measurement of the load current without introducing significant losses or disturbing the excitation signal. Experimental testing confirmed that the system delivers stable sinusoidal signals with consistent current sensing performance across the tested operating range.

The design prioritizes low cost, compact integration, and ease of reproducibility over high-end measurement accuracy, resulting in moderate current sensing deviations while maintaining sufficient performance for ELF applications. Overall, the proposed device represents a low-cost and versatile platform suitable for laboratory experimentation, educational use, and small-scale testing of inductive loads.

Ethics statements.

The authors have nothing to declare under this heading.

AI declaration statement.

During the preparation of this work, the authors used ChatGPT, to assist in generating the graphical abstract and Fig. 1. The authors reviewed and edited the output and take full responsibility for the content of the publication.

### CRediT authorship contribution statement

**Michal Labuda:** Writing – original draft, Validation, Supervision, Methodology, Formal analysis, Data curation. **Veronika Wohlmuthova:** Writing – review & editing, Visualization, Software. **Ladislav Janousek:** Writing – review & editing, Supervision, Formal analysis.

## Declaration of competing interest

The authors declare that they have no known competing financial interests or personal relationships that could have appeared to influence the work reported in this paper.
